# Indications for the use of metronidazole in the treatment of non-periodontal dental infections: a systematic review

**DOI:** 10.1093/jacamr/dlac072

**Published:** 2022-08-09

**Authors:** Lesley Cooper, Nikolai Stankiewicz, Jacqueline Sneddon, R Andrew Seaton, Andrew Smith

**Affiliations:** Scottish Antimicrobial Prescribing Group, Healthcare Improvement Scotland, Delta House, 50 West Nile Street, Glasgow, G1 2NP, UK; Damira Dental Studios, Head Office, First Floor, The High Barn Pinner Hill Farm, Pinner Hill, HA5 3YQ, UK; Scottish Antimicrobial Prescribing Group, Healthcare Improvement Scotland, Delta House, 50 West Nile Street, Glasgow, G1 2NP, UK; Scottish Antimicrobial Prescribing Group, Healthcare Improvement Scotland, Delta House, 50 West Nile Street, Glasgow, G1 2NP, UK; Queen Elizabeth University Hospital, NHS Greater Glasgow and Clyde, Govan Road, Glasgow, UK; College of Medical, Veterinary & Life Sciences, Glasgow Dental Hospital & School, University of Glasgow, 378 Sauchiehall Street, Glasgow, G2 3JZ, UK

## Abstract

**Background:**

Dental practitioners are the largest prescribers of metronidazole. Antibiotics should only be prescribed when systemic involvement is clear and should be limited to monotherapy with β-lactams in the first instance.

**Objectives:**

To determine whether metronidazole used as monotherapy or in addition to a β-lactam antibiotic offers any additional benefit over β-lactam monotherapy in non-periodontal dental infections.

**Methods:**

Searches of Ovid Medline, Ovid Embase, Cochrane library and trials registries, forward and backward citations, for studies published between database inception and 2 August 2021. All randomized clinical trials (RCTs) and non-randomized trials comparing either systemic metronidazole monotherapy or metronidazole combined with a β-lactam with β-lactam monotherapy for the treatment of non-periodontal dental infections in adults or children in outpatient settings were included.

**Results:**

Four publications reporting three RCTs comparing metronidazole with a β-lactam antibiotic were recovered. Studies were conducted in the 1970s–80s and aimed to demonstrate metronidazole was as effective as penicillin for the treatment of acute pericoronitis or acute apical infections with systemic involvement. Meta-analysis of results was not possible due to differences in measurement of infection signs. All studies concluded that metronidazole and penicillin are equally effective for the treatment of non-periodontal dental infections with systemic involvement.

**Conclusions:**

Metronidazole does not provide superior clinical outcomes (alone or in combination with a β-lactam) when compared with a β-lactam antibiotic alone for the treatment of non-periodontal dental infections in general dental practice. Guidelines should reinforce the importance of surgical interventions and if appropriate the use of a single agent narrow-spectrum β-lactam.

## Introduction

First-line management of non-periodontal dental infection without systemic symptoms is local treatment of the infected tooth to remove the source of inflammation or infection. In the majority of cases this is the only intervention required.^1,2^ If signs of systemic involvement are present, most international guidelines recommend monotherapy with a β-lactam antibiotic.^3–6^ There is currently equipoise with regards to choice of agent when combined with local intervention (drainage, root canal therapy and/or removal of the cause).^7^ As antibiotic use isa key driver of antimicrobial resistance it is important that prescribers adhere to guidelines and understand the underpinning evidence, only prescribing antibiotics when there are clear signs of systemic spread and likely benefit for the patient. Therefore when antibiotics are deemed necessary, WHO Access (narrow spectrum) antibiotics should be selected to minimize impact on antimicrobial resistance and given for the shortest possible duration to achieve clinical improvement. Recommended first-line antimicrobial treatment consists of phenoxymethylpenicillin 500–1000 mg or amoxicillin 500–1000 mg for up to 5 days with review at 3 days. Metronidazole should be considered as second choice for patients not allergic to penicillin or as an adjunct in severe infections.^4,6^

The Scottish Antimicrobial Prescribing Group (SAPG) leads the antimicrobial stewardship programme in Scotland and includes a focused dental subgroup comprising multidisciplinary representation from general dental practice, academia, national dental organizations and pharmacy. Whilst metronidazole is the second most commonly prescribed antibiotic in dental practice (after amoxicillin), dental use accounts for 59% of all primary care use of metronidazole in Scotland.^8^ Surveillance of prescribing in 2020 showed a 3.2% increase in dental antibiotic prescribing to 10.4% of antimicrobial prescriptions in primary care in Scotland as a result of restrictions on surgical interventions during COVID-19.^9^ The relatively high proportion of metronidazole prescribing in general dental practice suggests that guidance and behavioural science interventions will require review and updating. Furthermore, in a recent study comparing antibiotic prescribing patterns, dentists in England and Scotland prescribed approximately three to five times more antibiotics than those in Norway and Sweden and were more likely to prescribe metronidazole.^10^

The present study aimed to understand whether metronidazole used as monotherapy or in addition to a β-lactam antibiotic offers any additional benefit over β-lactam monotherapy in non-periodontal dental infections.

## Methods

### Literature search strategy

This systematic review is reported in accordance with the preferred reporting items for systematic reviews and meta-analysis (PRISMA) statement 2020.^11^ The objective, inclusion criteria and methods of analysis were specified in advance and published in a protocol (Prospero CRD42021269247).

### Inclusion/exclusion criteria

Studies included in the review had to adhere to inclusion criteria as listed in Table 1. Case reports, retrospective cohort studies, systematic reviews, animal trials, letters to editors, *in vivo* and *in vitro* studies were excluded. We also excluded studies with incomplete data collection; studies with no comparison group, or those that involved any additional therapy (other than analgesia) that could affect the outcomes; and studies that reported on inpatient treatment of patients with dental infections or prophylactic treatment.

**Table 1.** Inclusion criteria

	Criterion
1	RCTs, non-randomized trials
2	Human participants (adults and children) undergoing outpatient treatments for non-periodontal dental infections in general dental practices
3	Studies that reported comparisons of metronidazole alone or plus β-lactams with β-lactams only
4	Clearly defined clinical parameters according to which diagnosis and resolution of infection is made—e.g. clinician assessment of swelling and appearance of soft tissues, trismus and fever. Patient reported pain intensity evaluated by any validated measure e.g. numeric rating scale/visual analogue scale (VAS)
5	Studies that reported systemic antibiotics prescribed as adjuvant to surgical treatment
6	Studies that reported systemic antibiotics prescribed without surgical treatment

### Search strategy

To identify relevant publications we searched the Cochrane library, Ovid Embase and Ovid Medline electronic databases from inception to 2 August 2021. ClinicalTrials.gov and the WHO International Trial Registry Platform were searched for ongoing and unpublished studies. Backwards and forwards search of selected studies were conducted. The search strategy was developed in consultation with an information scientist; full search strategies for each database can be found in the Supplementary data (available at *JAC-AMR* Online).

### Data collection and analysis

Search results were collated on EndNote^™^ 20. Following removal of duplicates, all titles and available abstracts were screened by two authors (N.S. and L.C.). Disagreements were resolved by consensus or by discussion with a third author (J.S.). Full text was retrieved for all records deemed to meet the inclusion criteria. Data from eligible studies were extracted using a tool created for this study. The following information was extracted: author and year, study setting, study design, type of infection(s), type and dose of antibiotic used, duration of treatment, sample size in each group, follow-up period and reported clinical outcomes.

### Assessment of methodological quality

Studies selected for critical appraisal were assessed independently by two authors (L.C. and N.S.) using the Joanna Briggs Institute (JBI)^12^ tool for assessing risk of bias in randomized clinical trials (RCTs). Disagreements were resolved by consensus or by discussion with a third author (J.S.).

### Data synthesis

We planned to carry out meta-analysis, however this was not possible as although studies reported similar outcomes, for people with similar clinical conditions, methods of assessment used were heterogeneous and subjective. We therefore present a narrative review of included studies.

## Results

### Results of the search

**Figure 1.**
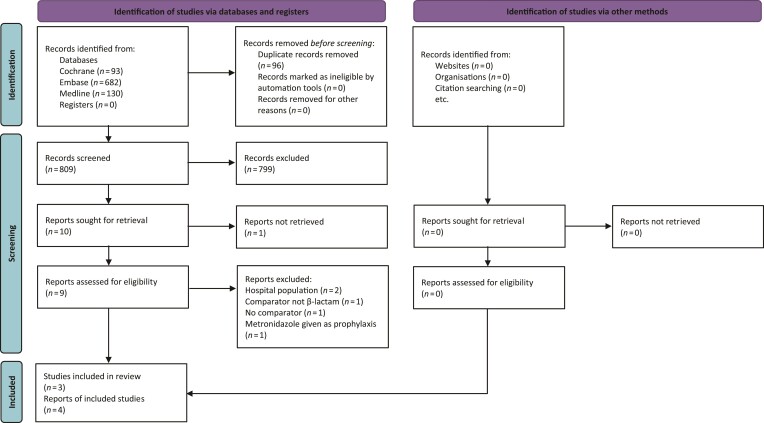
PRISMA flow chart. Adapted from: Page MJ, McKenzie JE, Bossuyt PM *et al.* The PRISMA 2020 statement: an updated guideline for reporting systematic reviews. *BMJ* 2021; **372**: n71 (CC BY 4.0). For more information, visit: http://www.prisma-statement.org/.

Following removal of duplicates, the electronic searches identified a total of 809 references. No additional trials were identified. Independent review of the titles and abstracts, where available, excluded 799 references from further analysis and resulted in retrieval of full text for nine potentially relevant publications, translated where required. One study could not be retrieved. Nine studies were evaluated against the inclusion criteria, five were excluded and reason for exclusion recorded. Four publications^13–16^ that reported three RCTs could be included. Figure 1 details the study selection flow chart.

### Risk of bias in included studies

Agreed results of independent author judgements about the risk of bias in included studies are presented in Table 2. Three per protocol studies were appraised. Risk of selection bias was considered to be low in two studies^15,16^ and unclear in the third.^13^ Two studies were considered to have adequate concealment of allocation,^15,16^ this was unclear in the third.^13^ Two studies were judged to have adequate blinding of participants and low risk of detection bias^15,16^ while risk was unclear in the third study.^13^ Risk of detection bias was deemed low in all three included studies.

**Table 2.** Critical appraisal of studies

Question	Hood 1978^13^	McGowan 1977^15^	Meiss 1982^16^
1. Was true randomization used for assignment of participants to treatment groups?	Y	Y	UC
2. Was allocation to treatment groups concealed?	N	Y	Y
3. Were treatment groups similar at baseline?	Y	Y	Y
4. Were participants blind to treatment assignment?	UC	Y	Y
5. Were those delivering treatment blind to treatment assignment?	UC	Y	Y
6. Were outcomes assessors blind to treatment assignment?	UC	Y	UC
7. Were treatment groups treated identically other than the intervention of interest?	N	Y	Y
8. Was follow-up complete and if not, were differences between groups in terms of their follow up adequately described and analysed?	Y	UC	Y
9. Were participants analysed in the groups to which they were randomized?	Y	Y	Y
10. Were outcomes measured in the same way for treatment groups?	Y	Y	Y
11. Were outcomes measured in a reliable way?	Y	Y	Y
12. Was appropriate statistical analysis used?	UC	Y	Y
13. Was the trial design appropriate, and any deviations from the standard RCT designed accounted for in the conduct and analysis of the trial?	Y	Y	Y
Total	7	12	11

Y, yes; N, no; N/A, not applicable; UC, unclear.

### Description of studies

Three randomized controlled trials satisfied the inclusion criteria.^13,15,16^ Details for individual studies are reported in Table 3. Two studies were conducted in the UK, in a dental school^13^ and a dental hospital,^15^ and the third was conducted in a dental faculty in Argentina.^16^ The study samples comprised 37,^13^ 31^15^ and 80^16^ participants suffering from acute pericoronitis or acute apical infection. All studies were of parallel group design. Two had two arms;^13,15^ both compared the effectiveness of oral metronidazole monotherapy 200 mg 8 hourly for 3 days^13^ or 200 mg 6 hourly for 5 days^15^ with β-lactam monotherapy consisting of: one dose of penicillin G given intramuscularly (IM) followed by oral penicillin V 250 mg four times per day for 5 days^13^ or oral penicillin V 250 mg four times per day for 5 days plus paracetamol as required.^15^ The third study^16^ compared oral metronidazole monotherapy 250 mg 8 hourly for 4 days in a four-arm study with oral amoxicillin 500 mg, oral erythromycin 500 mg or doxycycline 50 mg monotherapy administered three times per day for 4 days.^16^ All studies reported on objective and subjective clinician assessed and/or patient reported improvement in signs and symptoms including facial swelling, pain, fever, inflammation of gingival mucosa, trismus, presence of pus and cervical adenopathy. Two studies asked patients to report use of analgesia.^15,16^ Patients were reviewed daily for 3 days,^13^ on Days 1, 2 and 7^15^ or 24, 48 and 72 h after first visit.^16^

**Table 3.** Study characteristics

First author and year/Setting	Design; type of infection; length of study	Intervention group sample size; type and dose of antibiotic used and duration of treatment	Control group sample size; type and dose of antibiotic used and duration of treatment	Clinical outcomes
Hood 1978^13^ Dental school UK	RCT; acute dental infection including acute pericoronitis and acute apical infection; 3 days—patients seen daily	*N *= 18 (5 acute pericoronitis and 12 apical infections); metronidazole 200 mg 8 hourly for 3 days	*N *= 19 (7 acute pericoronitis and 12 apical infection); penicillin G 600 mg IM immediately + penicillin V 250 mg four times/day for 5 days	A marked clinical improvement was apparent within 24–48 h of commencing treatment in both groups. Metronidazole appeared to be as effective as penicillin
McGowan 1977^15^ Dental hospital UK	RCT; acute pericoronitis; 7 days with review on Day 1 or 2 to exclude need for more intensive therapy	*N *= 13; metronidazole 200 mg four times/day for 5 days + 500 mg paracetamol as required	*N *= 18; phenoxymethylpenicillin 250 mg four times/day for 5 days + 500 mg paracetamol as required	All patients reported improvement in symptoms at final visit. *n *= 6 in metronidazole and *n *= 8 in penicillin group reported no pain. Reduction in clinical signs was noted. *n *= 3 patients on metronidazole and *n *= 3 on penicillin reported side effects e.g. nausea, constipation and tiredness. No significant between group differences in any measures including amount of analgesia taken
Meiss 1982^16^ Faculty of dentistry Argentina	RCT; acute pericoronitis; 3 days. Review at 24, 48 and 72 h after first visit	*N *= 20; metronidazole 250 mg 8 hourly for 4 days + 500 mg aspirin as required	*N *= 20; amoxicillin 500 mg 8 hourly for 4 days + aspirin	All pain had resolved in the amoxicillin and metronidazole groups at 72 h. In the erythromycin and doxycycline groups pain persisted at 72 h. Facial swelling was statistically significantly improved in metronidazole and amoxicillin treatment groups compared with erythromycin and doxycycline groups. At 72 h 55% of patients who received amoxicillin and 60% on metronidazole no longer had swelling or flushing in the soft tissues neighbouring the lesion. In the erythromycin and doxycycline groups only 10% no longer had swelling or flushing in the soft tissues. Limitation of mandibular opening was resolved at 72 h in the amoxicillin and metronidazole group and persisted in 15% and 18%, respectively, in the erythromycin and doxycycline groups
			*N *= 20; erythromycin 500 mg 8 hourly for 4 days + aspirin	
			*N *= 20; doxycycline 50 mg 8 hourly for 4 days + aspirin	

### Effects of interventions

All studies reported improvement in clinical features. Hood^13^ provided an overall summary that there was a marked clinical improvement within 24–48 h of commencing treatment in both groups. In McGowan’s^15^ study all patients reported improvement in symptoms and reduction in clinical signs were noted. No significant between group differences were reported. Meiss^16^ reported that all pain had resolved in the amoxicillin and metronidazole groups at 72 h and persisted in the erythromycin and doxycycline groups. Facial swelling, flushing in the soft tissues and mandibular opening were also improved in patients prescribed amoxicillin and metronidazole compared with patients in the erythromycin and doxycycline groups. All studies concluded equal efficacy of metronidazole and penicillin.

## Discussion

In the context of managing dental infections in a general dental practice setting, evaluation of the studies included in this review suggests that metronidazole either alone or in combination does not provide superior clinical outcomes in terms of resolution or symptom relief when compared with a β-lactam antibiotic for the treatment of non-periodontal dental infections. This supports recommendations in current guidelines that suggest a β-lactam antibiotic should be used first line.^2–6^ Failure to resolve the infection should prompt a re-evaluation of signs and symptoms and if clinically appropriate repeat surgical interventions. We do not consider the inpatient management of patients presenting with sepsis and severe odontogenic infections that usually require IV antibiotics with incision and drainage under general anaesthesia.

The microbiota associated with dentoalveolar infections commonly comprise classic pathogens associated with the oral flora, such as the *Streptococcus anginosus* group and obligate anaerobes such as *Prevotella*, *Fusobacteria* and Gram-positive anaerobic cocci species.^17^ It should be noted though that with the advent of molecular techniques and improvement in anaerobic culture methodologies this is a qualitative over-simplification of the range of bacterial species involved in intra-oral infections.^18^ However, from a practical clinical perspective the available literature on clinical outcomes suggests that antimicrobial strategies that target the common classic pathogens associated with intra-oral infections provides a sufficient safety profile for management. In terms of antimicrobial susceptibility the *S. anginosus* group are universally susceptible to narrow spectrum β-lactams such as penicillin V and inherently resistant to metronidazole. The incidence of β-lactam resistance in the obligate anaerobes associated with intra-oral infections is difficult to quantify with certainty due to the paucity of robust susceptibility testing i.e. consistent test methodology/breakpoints used and linked epidemiological data. A similar case could be made for an absence of accurate determinations of metronidazole resistance in obligate anaerobic populations, although workers have noted the appearance of metronidazole resistance in microbial species that can also be recovered from dental infections.^19,20^ It is therefore challenging to empirically determine the clinical importance of antimicrobial susceptibility profiles in patients treated for dental infections in a general dental practice setting as the critical treatment element is the surgical removal of the source of infection. Removal of the source of infection effectively reduces the microbial challenge and virulence potential of the polymicrobial infection.

It is unclear from this and other reviews on the use of metronidazole in dental practice why there has been a trend for increased use of metronidazole, usually in the form of dual prescribing with a β-lactam agent to the extent that dental practitioners are now the largest prescribers of metronidazole in healthcare.^9^ There is no evidence to support this level of use in the management of dental infections in general dental practice. If antibiotics are required for the management of infection, then in line with good antimicrobial stewardship principles, the use of a single agent narrow spectrum β-lactam should be first choice. Evidence (such as it is) suggests that penicillin V appears to fit the criteria of a narrow-spectrum agent with good clinical efficacy whilst covering the majority of the classic pathogens involved in dental infections.^4^ Metronidazole on the other hand with activity limited to obligate anaerobes would not target the *S. anginosus* group and may also have disproportionate adverse effects on the commensal microbiota, so should be used more sparingly^21,22^ although collecting and comparing findings across studies is challenging. To support behaviour change in metronidazole prescribing in general dental practice the use of prescribing audits at a local level would be helpful to understand prescribing practice. Qualitative research using interviews or focus groups could also be considered to gain more insight around prescribers’ attitudes and behaviours in selecting antibiotic treatments.^23^

In conclusion, metronidazole does not provide superior clinical results when compared with a β-lactam antibiotic for the treatment of non-periodontal dental infections. It should be noted that these conclusions are based on limited clinical trial evidence, with none more recent than 1982, this highlights the need for more current trials on antimicrobial efficacy for the management of dental infections. Guidelines and behavioural sciences should reinforce the importance of surgical interventions and if antimicrobials are required the use of a single agent narrow-spectrum antibiotic, such as phenoxymethylpenicillin, recommended. Microbiological and clinical surveillance schemes should be improved to monitor the antimicrobial susceptibilities and clinical outcomes of patients with severe odontogenic infections as these may be the first indicators of clinically significant changes in the antimicrobial resistance patterns in the oral flora.

## Supplementary Material

dlac072_Supplementary_Data

## Supplementary data

The search strategy is available as Supplementary data at *JAC-AMR* Online.

## References

[1] Ellison SJ . The role of phenoxymethylpenicillin, amoxicillin, metronidazole and clindamycin in the management of acute dentoalveolar abscesses-a review. Br Dent J 2009; 206: 357–62. 19357666 10.1038/sj.bdj.2009.257

[2] Teoh L , Cheung MC, Dashper S et al Oral antibiotic for empirical management of acute dentoalveolar infections-a systematic review. Antibiotics 2021; 10: 240–56. 33670844 10.3390/antibiotics10030240 PMC7997333

[3] NICE . Summary of Antimicrobial Prescribing Guidance – Managing Common Infections, August 2021. https://www.bnf.org/wp-content/uploads/2021/09/summary-antimicrobial-prescribing-guidance_aug-21_final.pdf.

[4] Scottish Dental Clinical Effectiveness Programme . Drug Prescribing for Dentistry, June 2021 Update, 3rd Edition. https://www.sdcep.org.uk/media/ckgfnx3w/sdcep-drug-prescribing-ed-3-update-june-2021.pdf.

[5] Segura-Egea JJ , Gould K, Şen BH et al European Society of Endodontology position statement: the use of antibiotics in endodontics. Int Endod J 2018; 51: 20–25. 28436043 10.1111/iej.12781

[6] Palmer NO . Antimicrobial Prescribing in Dentistry: Good Practice Guidelines, 3rd Edition. Faculty of General dental Practice (UK) and the Faculty of Dental Surgery, 2020. https://cgdent.uk/wp-content/uploads/2021/08/Antimicrobial-Prescribing-in-Dentistry-2020-online-version.pdf.

[7] Martins JR , Chagas OL Jr, Velasques BD et al The use of antibiotics in odontogenic infections: what is the best choice? A systematic review. J Oral Maxillofac Surg 2017; 75: 2606.e1–06.e11. 28893540 10.1016/j.joms.2017.08.017

[8] National Services Scotland . Scottish One Health Antimicrobial Use and Antimicrobial Resistance in 2019: Annual Report, Revised 7 September 2021. https://www.nss.nhs.scot/publications/scottish-one-health-antimicrobial-use-and-antimicrobial-resistance-in-2019/.

[9] ARHAI Scotland . Scottish One Health Antimicrobial Use and Antimicrobial Resistance Report 2020. ARHAI Scotland, 2021. https://www.nss.nhs.scot/publications/scottish-one-health-antimicrobial-use-and-antimicrobial-resistance-in-2020/.

[10] Smith A , Al-Mahdi R, Malcolm W et al Comparison of antimicrobial prescribing for dental and oral infections in England and Scotland with Norway and Sweden and their relative contribution to national consumption 2010-2016. BMC Oral Health 2020; 20: 172. 32546149 10.1186/s12903-020-01163-x PMC7298788

[11] Page MJ , McKenzie JE, Bossuyt PM et al The PRISMA 2020 statement: an updated guideline for reporting systematic reviews. BMJ 2021; 372: n71. 33782057 10.1136/bmj.n71 PMC8005924

[12] Joanna Briggs Institute . Critical Appraisal Tools Australia. https://jbi.global/critical-appraisal-tools.

[13] Hood FJC . The place of metronidazole in the treatment of acute oro-facial infection. J Antimicrob Chemother 1978; 4: 71–3. 99425 10.1093/jac/4.suppl_c.71

[14] Ingham HR , Hood FJ, Bradnum P et al Metronidazole compared with penicillin in the treatment of acute dental infections. Br J Oral Surg 1977; 14: 264–69. 321005 10.1016/0007-117x(77)90035-x

[15] McGowan DA , Murphy KJ, Sheiham A. Metronidazole in the treatment of severe acute pericoronitis. A clinical trial. Br Dent J 1977; 142: 221–3. 403925 10.1038/sj.bdj.4803898

[16] Meiss A , Caride ER. [Evaluation of the efficiency of metronidazole as chemotherapy for acute pericoronitis.] [Spanish]. Rev Asoc Odontol Argent 1982; 70: 93–6. 6763727

[17] Robertson D , Smith AJ. The microbiology of the acute dental abscess. J Med Microbiol 2009; 58: 155–62. 19141730 10.1099/jmm.0.003517-0

[18] Siqueira JF , Rocas IN. Microbiology and treatment of acute periapical abscesses. Clin Microbiol Rev 2013; 26: 255–73. 23554416 10.1128/CMR.00082-12 PMC3623375

[19] Veloo AC , Boiten KE, Wekema-Mulder GJ. Antibiotic susceptibility profiles of *Prevotella* species in the Netherlands. Int J Antimicrob Agents 2015; 45: 554–6. 25726442 10.1016/j.ijantimicag.2015.01.003

[20] Veloo AC , Welling GW, Degener JE. Antimicrobial susceptibility of clinically relevant Gram-positive anaerobic cocci collected over a three-year period in the Netherlands. Antimicrob Agents Chemother 2011; 55: 1199–203. 21189338 10.1128/AAC.01771-09 PMC3067104

[21] Jakobsson HE , Jernberg C, Andersson AF et al Short-term antibiotic treatment has differing long-term impacts on the human throat and gut microbiome. PLoS One 2010; 5: e9836. 20352091 10.1371/journal.pone.0009836 PMC2844414

[22] Adamsson I , Edlund C, Sjöstedt S et al Comparative effects of cefadroxil and phenoxymethylpenicillin on the normal oropharyngeal and intestinal microflora. Infection 1997; 25: 154–8. 9181382 10.1007/BF02113603

[23] Thompson W , Tonkin-Crine S, Pavitt SH et al Factors associated with antibiotic prescribing for adults with acute conditions: an umbrella review across primary care and a systematic review focusing on primary dental care. J Antimicrob Chemother 2019; 74: 2139–52. 31002336 10.1093/jac/dkz152 PMC6640312

