# Supplementary material for: Indications for the use of metronidazole in the treatment of non-periodontal dental infections: a systematic review
Source: JAC Antimicrob Resist. 2022 Aug 9;4(4):dlac072. doi: 10.1093/jacamr/dlac072 (PMC9361036; doi:10.1093/jacamr/dlac072)

**Supplementary data**

**Search strategy**

***Cochrane Library***

Key words searched: Tooth or teeth or molar or incisor or cuspid or bicuspid or dental; bacterial infections; periapical abscess or periodontal abscess; pericoronitis; odontogenic infection; metronidazole; betalactams n=93

***Ovid Embase***


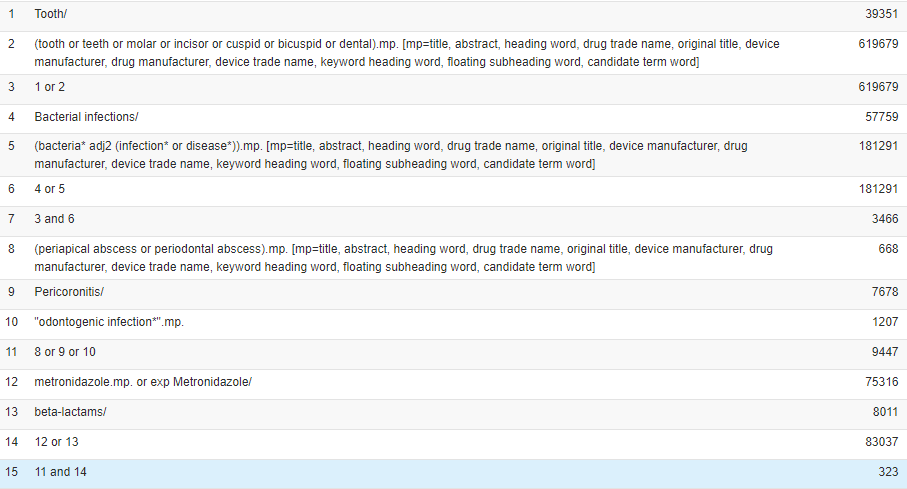


***Ovid Medline***


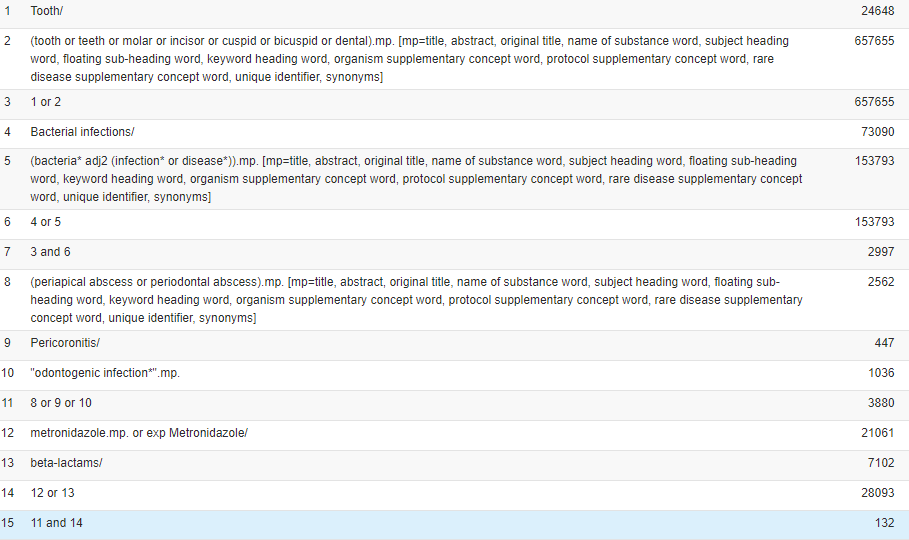

Supplement: dlac072_Supplementary_Data [file dlac072_supplementary_data.docx]
